# Seawater physics and chemistry along the Med-SHIP transects in the Mediterranean Sea in 2016

**DOI:** 10.1038/s41597-023-02835-3

**Published:** 2024-01-09

**Authors:** Katrin Schroeder, Vedrana Kovačević, Giuseppe Civitarese, Dimitris Velaoras, Marta Álvarez, Toste Tanhua, Loïc Jullion, Laurent Coppola, Manuel Bensi, Laura Ursella, Chiara Santinelli, Michele Giani, Jacopo Chiggiato, Mohamed Aly-Eldeen, Georgia Assimakopoulou, Giancarlo Bachi, Boie Bogner, Mireno Borghini, Vanessa Cardin, Marin Cornec, Antonia Giannakourou, Louisa Giannoudi, Alexandra Gogou, Melek Golbol, Or Hazan, Clarissa Karthäuser, Martina Kralj, Evangelia Krasakopoulou, Frano Matić, Hrvoje Mihanović, Stipe Muslim, Vassilis P. Papadopoulos, Constantine Parinos, Anne Paulitschke, Alexandra Pavlidou, Elli Pitta, Maria Protopapa, Eyal Rahav, Ofrat Raveh, Panagiotis Renieris, Nydia C. Reyes-Suarez, Eleni Rousselaki, Jacop Silverman, Ekaterini Souvermezoglou, Lidia Urbini, Christina Zeri, Soultana Zervoudaki

**Affiliations:** 1 Consiglio Nazionale delle Ricerche-Istituto di Scienze Marine (CNR-ISMAR), Venice and La Spezia, Italy; 2 National Institute of Oceanography and Applied Geophysics - OGS, Trieste, Italy; 3 Hellenic Centre for Marine Research, HCMR, Institute of Oceanography, Anavyssos, Greece; 4 Instituto Español de Oceanografía, IEO-CSIC A Coruña, Spain; 5 GEOMAR Helmholtz Centre for Ocean Research Kiel, Kiel, Germany; 6 Sorbonne Université, CNRS, Laboratoire d’Océanographie de Villefranche, Villefranche-sur-Mer, France; 7 Sorbonne Université, CNRS, OSU STAMAR, UAR2017, 4 Place Jussieu, 75252, Paris, cedex 05 France; 8 Consiglio Nazionale delle Ricerche-Istituto di Biofisica (CNR-IBF), Pisa, Italy; 9 National Institute of Oceanography and Fisheries, NIOF, Egypt EG,; 10 Israel Oceanographic and Limnological Research, IOLR, Haifa, Israel; 11 University of the Aegean, Department of Marine Sciences, Mytilene, Greece; 12 University Department of Marine Studies, University of Split, Split, Croatia; 13 Institute of Oceanography and Fisheries, Split, Croatia

**Keywords:** Physical oceanography, Marine chemistry

## Abstract

The Mediterranean Sea has been sampled irregularly by research vessels in the past, mostly by national expeditions in regional waters. To monitor the hydrographic, biogeochemical and circulation changes in the Mediterranean Sea, a systematic repeat oceanographic survey programme called Med-SHIP was recommended by the Mediterranean Science Commission (CIESM) in 2011, as part of the Global Ocean Ship-based Hydrographic Investigations Program (GO-SHIP). Med-SHIP consists of zonal and meridional surveys with different frequencies, where comprehensive physical and biogeochemical properties are measured with the highest international standards. The first zonal survey was done in 2011 and repeated in 2018. In addition, a network of meridional (and other key) hydrographic sections were designed: the first cycle of these sections was completed in 2016, with three cruises funded by the EU project EUROFLEETS2. This paper presents the physical and chemical data of the meridional and key transects in the Western and Eastern Mediterranean Sea collected during those cruises.

## Background & Summary

### Introduction and scientific objectives

The ocean is variable and plays a significant role in the Earth’s energy and freshwater balance^1^, but it is poorly sampled, particularly below 2000 m. The Mediterranean Sea is a marginal sea that plays a significant role in the regional freshwater, heat and carbon budget. It is often described as a miniature ocean, where typical oceanic processes (such as dense water formation, mesoscale dynamics, thermohaline circulation) occur faster and on smaller scales than in the open ocean [e.g.^2^]. The Mediterranean Sea has also experienced remarkable changes in its physical and biogeochemical properties over the past decades, such as the Eastern Mediterranean Transient event (see next section “Geographical and oceanographic settings”), the increase of salinity and temperature in the deep layers, and the decrease of dissolved oxygen concentrations in the intermediate waters. These changes have important implications for the climate, ocean biogeochemistry, and ecosystem functioning of the region.

To observe and understand these changes, regular surveys of the Mediterranean circulation with comprehensive physical and biogeochemical measurements are needed. However, in the past, the Mediterranean has been sampled sporadically in time and space, mostly by national expeditions in regional waters. The first basin wide zonal transect has been described by^3^, in his early studies of Mediterranean water masses. During the 1980s and 1990s four large international programs, i.e. the Gibraltar Experiment (GIBEX^4^), the Physical Oceanography of the Eastern Mediterranean (POEM^5^), the Western Mediterranean Circulation Experiment (WMCE^6^), and the Programme de Recherche International en Mediterranée Occidentale (PRIMO^7^), set the baseline to a thorough definition of the main Mediterranean Sea characteristics. We particularly note the pioneering studies of Wolfgang Roether^8–12^, who used observations of chemical transient tracers (e.g., chlorofluorocarbons, tritium), that provided insights on ventilation rates, water mass formation processes of the Mediterranean Sea and the Eastern Mediterranean Transient event. Since the end of these programs, uncoordinated ship-based research efforts, driven mainly by national interests, provided fragmented and sporadic observations.

**Fig. 1:**
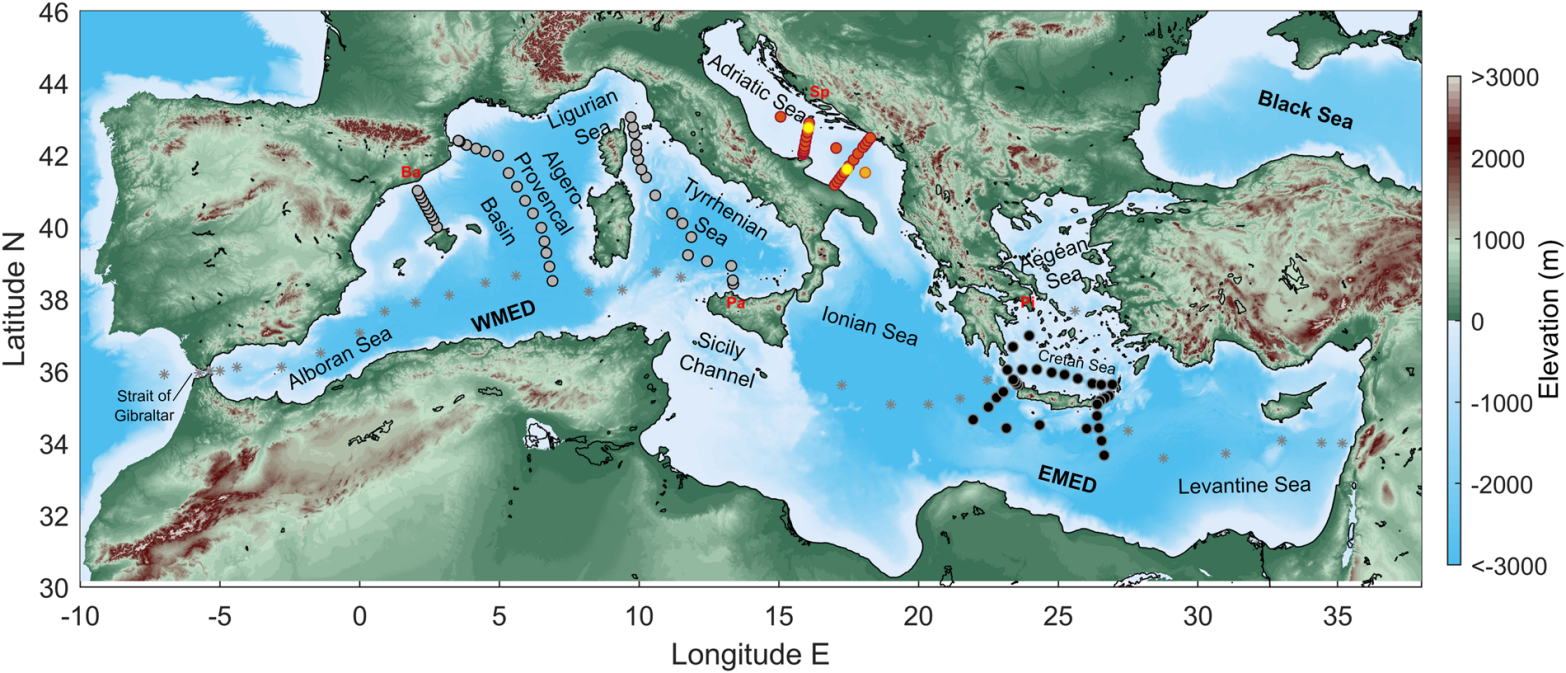
Station map of the Med-SHIP cruises: TAlPro2016 (grey circles), CRELEV2016 (black circles), ESAW2015 + ESAW2016 common stations in red (ESAW2015 only in orange and ESAW2016 only in yellow). The ports of these cruises are indicated in red letters (Pa = Palermo, Ba = Barcelona, Pi = Piraeus, Sp = Split). For completeness, MED01 GO-SHIP stations (repeated in 2011 and 2018, not presented in this paper) are shown by light grey stars. All cruises are ideally performed coast-to-coast, however permissions to work in southern and easternmost shore waters are rarely given. For schematics of main currents refer to e.g.^111^.

Marginal seas such as the Mediterranean Sea and others were not originally considered in the international GO-SHIP (Global Ocean Ship-based Hydrographic Investigations Program) or in global data synthesis efforts such as GLODAP^13,14^. In 2011, an outcome of the 43rd CIESM (Mediterranean Science Commission) Workshop in Supetar (Croatia) was the recommendation for repeat oceanographic surveys within the Mediterranean Sea, in a programme called Med-SHIP^15^. Similarly to the international GO-SHIP programme for the global ocean, Med-SHIP aims to detect the impact of climate change in the Mediterranean Sea by comprehensive observations of physical and biogeochemical variables. These variables should be measured to the highest international standards on a regular basis along a number of zonal and meridional hydrographic sections following the GO-SHIP guidelines^16^. There are two primary objectives for the Med-SHIP repeat hydrography cruises: (i) to observe long-term changes in physical and biogeochemical properties and (ii) to observe changes in the thermohaline circulation, both in the Mediterranean Sea as a whole, but also on sub-basin scales. Therefore, the Med-SHIP program consists of a zonal section from the Strait of Gibraltar to the easternmost Mediterranean and a number of meridional or key sections in the Eastern and Western Mediterranean basins (Fig. 1). The zonal survey is already officially part of the GO-SHIP reference sections, named as MED01 line (shown with light grey stars in Fig. 1), with the CTD and bottle data available at CCHDO^17^ and biogeochemical data included in the GLODAPv2 (Global Ocean Data Analysis Project) data product^18^. The western and eastern sections of Med-SHIP, the subject of this paper, are in the process of being proposed to GO–SHIP as “associated lines”.

This paper is the culmination of efforts in 2016 to obtain the first quasi-synoptic meridional surveys of the western and eastern Mediterranean Sea as part of MED-SHIP. Scientists from eight countries worked on three national research vessels to complete these cruises. The data reported here include temperature, salinity, pressure, inorganic nutrients, dissolved oxygen, total alkalinity, dissolved inorganic carbon, chlorophyll-a, pH, and chlorofluorocarbons (details on measured variables are given in Tables 1, 2). The results from these cruises adhere to the data policy of GO-SHIP, i.e., all data are published in open and free data repositories. The data are curated by specialized data centres, which are listed in Table 3 of section “Data Records”. The Med-SHIP programme also strives to achieve the accuracy and precision requirements articulated by GO-SHIP^19^.

**Table 1:** Details (parameter name, short name, unit, method, cruise) on the parameters measured by the CTD multiparametric probe and underway during each cruise.

Parameter name	Short name	Unit	Method	Cruise
date/time	Date/time	UTC		TalPro2016 CRELEV2016 ESAW
latitude/longitude	Latitude/ Longitude	degrees North/degrees East		TalPro2016 CRELEV2016 ESAW
pressure, water	Press	dbar	CTD, SBE 911+	TalPro2016 CRELEV2016 ESAW
temperature, water	Temp	°C	CTD, SBE 911+	TalPro2016 CRELEV2016 ESAW
salinity	Sal		CTD, SBE 911+	TalPro2016 CRELEV2016 ESAW
dissolved oxygen	O_2_	μmol kg^−1^	CTD with a SBE 43 calibrated using Winkler titration (except for ESAW)	TalPro2016 CRELEV2016 ESAW
fluorescence, chlorophyll	F chl	μg L^−1^ mg m^−3^	WET Labs ECO-AFL/FL fluorometer Chelsea AquaTracka III WET Labs ECO-AFL/FL fluorometer	TAlPro2016 CRELEV2016 ESAW
turbidity beam transmission	Turbidity BT	NTU%	WET Labs turbidimeter Chelsea AlphaTracka Mk II WET Labs transmissometer	TAlPro2016 CRELEV2016 ESAW
current velocity east-west	U	m s^−1^	lowered acoustic doppler current profiler (LADCP)	TAlPro2016 CRELEV2016
current velocity north-south	V	m s^−1^	lowered acoustic doppler current profiler (LADCP)	TAlPro2016 CRELEV2016
current velocity east-west	U	m s^−1^	vessel-mounted acoustic doppler current profiler (VM-ADCP)	TalPro2016 CRELEV2016 ESAW
current velocity north-south	V	m s^−1^	vessel-mounted acoustic doppler current profiler (VM-ADCP)	TalPro2016 CRELEV2016 ESAW
temperature, water	Temp	°C	SBE 21 Seacat Thermosalinograph	TAlPro2016, CRELEV2016
salinity, water	Sal		SBE 21 Seacat Thermosalinograph	TalPro2016 CRELEV2016

**Table 2:** Details (parameter name, short name, unit, method, cruise) on the parameters measured on discrete samples during each cruise.

Parameter name	Short name	Unit	Method	Cruise
dissolved oxygen	O_2_	μmol kg^−1^	Winkler titration	TalPro2016 CRELEV2016 ESAW
salinity	Sal		Salinometer	TalPro2016 CRELEV2016 ESAW
nitrate	NO_3_^-^	μmol kg^−1^	Autoanalyzer	TalPro2016 CRELEV2016 ESAW
nitrite	NO_2_^-^	μmol kg^−1^	Autoanalyzer	TalPro2016 CRELEV2016 ESAW
ammonium	NH_4_^+^	μmol kg^−1^	Autoanalyzer	ESAW
phosphate	PO_4_^3−^	μmol kg^−1^	Autoanalyzer	TalPro2016 CRELEV2016 ESAW
silicate	Si(OH)_4_	μmol kg^−1^	Autoanalyzer	TAlPro2016 CRELEV2016
dissolved inorganic carbon	DIC	μmol kg^−1^	Coulometric titration	TAlPro2016 CRELEV2016
total alkalinity	TA	μmol kg^−1^	Potentiometric titration in an open cell	TalPro2016 CRELEV2016 ESAW
pH	pH	total scale at 25 °C	Single beam spectrophotometer, un-purified dye Single beam Spectrophotometer, purified dye	TAlPro2016 CRELEV2016 ESAW
carbonate	CO_3_^2−^	μmol kg^−1^	Single beam spectrophotometer, Pb(ClO_4_)_2_	TAlPro2016
chlorofluorocarbon (CCl_2_F_2)_	CFC-12	pmol kg^−1^	Purge and trap Gas-Chromatograph with an Electron Capture Detector system	TalPro2016 CRELEV2016 ESAW2016
sulfur hexafluorid	SF_6_	fmol kg^−1^	Purge and trap Gas-Chromatograph with an Electron Capture DetectorGC-ECD system	TalPro2016 CRELEV2016 ESAW2016
dissolved organic carbon	DOC	μmol kg^−1^	High Temperature Catalytic Oxidation	CRELEV2016 ESAW
total dissolved phosphorus and nitrogen	TDP and TDN	µmol kg^−1^	Wet chemical oxidation and automated segmented flow colorimetric analysis	CRELEV2016 ESAW

**Table 3:** For each cruise all necessary information on data access (citations) are listed.

Cruise name (dates)	physics (CTD, Conductivity - Temperature - Depth probe)	chemistry (BTL, bottle)	LADCP (Lowered Acoustic Doppler Current Profiler)	VM-ADCP (Vessel-mounted Acoustic Doppler Current Profiler)	thermo-salinograph	NCEI OCADS link (National Centers for Environmental Information Ocean Carbon and Acidification Data Portal)	CSR link (Cruise Summary Report)
TAlPro2016 (19.08.2016-28.08.2016)	^83^	^84^	^85^	^86^	^87^	^88^	https://csr.seadatanet.org/report/20166062
ESAW2015 (10.12.2015-16.12.2015)	^90^	^91^	not collected	^92^	not collected	not participating	https://nodc.ogs.it/catalogs/csrdetails/2774?3
ESAW2016 (05.04.2016-10.04.2016)	^93^	^94^	not collected	^95^	not collected	not participating	https://nodc.ogs.it/catalogs/csrdetails/2191?2
CRELEV2016 (02.06.2016-10.06.2016)	^96^	^89^	https://csr.seadatanet.org/report/20165899				

The last column contains the link to the cruise summary reports (CSR), which describe the cruise details, but do not contain any data.

In 2016, the meridional sections in the Western Mediterranean Sea (WMED) were carried out during the TAlPro2016 cruise (*T*yrrhenian and *Al*gero-*Pro*vencal cruise; stations are shown by grey dots in Fig. 1^20^). The meridional and key sections in the Eastern Mediterranean Sea (EMED) were carried out during the CRELEV2016 cruise (*Cre*tan Sea and *Lev*antine cruise; stations are shown by black dots in Fig. 1^21^), and during the ESAW cruises (*E*volution and *S*preading of the Southern *A*driatic *W*aters cruises; stations are shown by yellow-orange-red dots in Fig. 1^22^), respectively. For the sake of clarity, it is necessary to note that the ESAW2016 cruise (called “ESAW leg 2” in the submitted datasets) was the second leg of a broader programme that included a first leg in December 2015, ESAW2015 (ESAW leg 1 in the submitted datasets). The aim was to conduct repeated measurements in the same area during two different periods (pre- and post- winter convection phases) and this is the reason why also one 2015 cruise is included in this paper.

In addition to the cruises we report on here, it is worth noting that other Med-SHIP sections have been repeated or are planned for future years: the trans-Mediterranean zonal Med-SHIP section (MED01 line) was repeated in 2011 and 2018^17,23^ and is planned again for 2025 or 2026; while the western meridional sections were recently repeated in spring 2022 (cruise TAlPro2022^24^).

The Med-SHIP program also aims to foster and facilitate regional collaboration between nations along the northern shores and countries in the Middle East and North Africa. This commitment is underscored by the rich diversity within our cruise teams and the authors of this paper, reflecting a wide geographical spectrum. Central to the Med-SHIP mission is capacity building, collaborative efforts, and knowledge exchange, which serve as vital mechanisms for bridging the research and expertise gap that exists between the northern shore countries and those in the Middle East and North Africa.

### Geographical and oceanographic settings

The Algero-Provençal basin is the main site of Western Mediterranean Deep Water (WMDW) formation driven by intense heat loss from the sea in the northern part of the basin. Since 2005 it became evident that the gradual temperature and salinity trends in the WMDW were interrupted by an abrupt shift towards higher temperature and salinity^25^. The onset of this shift, called the Western Mediterranean Transition, has been a major dense water formation event in winter 2004/2005 in the northern part of the WMED. Gradually this anomaly spread from its formation region and filled up the basin interior. The Tyrrhenian Sea acts as a blender of various water masses originating from the WMED and EMED^12,26,27^ and therefore plays an important role in the preconditioning the water column leading to the formation of WMDW^28,29^. Fresh and warm Atlantic Water (AW) enters the WMED via Gibraltar as an eastward boundary current along the African coast referred to as the Algerian Current^30^. This current, subject to strong mesoscale variability leading to the generation of long-lived anticyclonic eddies^27,31^, separates in a weak branch entering the Tyrrhenian Sea and a stronger branch entering the EMED through the Sicily Channel. The surface circulation along the northern slope of the Algero-Provençal basin is dominated by the Northern Current^27^ characterised by a strong seasonal variability in its mesoscale activity.

The return flow from the EMED towards the WMED is made of warm and salty Eastern Intermediate Water (EIW): this term refers generally to a mixture of intermediate water masses formed in the Levantine Sea (also known as Levantine Intermediate Water, LIW), and in the Cretan Sea (also known as Cretan Intermediate Water, CIW). The EIW crosses the Sicily Channel and is topographically constrained to veer north along the western coast of Sicily into the Tyrrhenian Sea. The Adriatic Sea used to be the main source of dense water for the whole EMED (i.e.^32,33^), and is also strongly influenced by climate change affecting the Mediterranean region^34^. The increase in temperature and salinity observed in the Adriatic Sea is due to processes that are not only local and have strong effects on the renewal and oxygenation of the deep waters throughout the Mediterranean Sea^35,36^. In the early 1990s, the Eastern Mediterranean Transient event took place, marking a shift in the dominant deep water source from the Adriatic Sea to the Aegean and Cretan Sea, characterized by massive overflows of dense (salty, but warm) waters^8,37^. After 2002 the Adriatic deep water formation restarted, and the recent Adriatic dense waters are warmer and saltier than before the Eastern Mediterranean Transient^38–42^. The Cretan Sea, however, remains a crucial region, which participates in Mediterranean thermohaline circulation with episodic and limited dense water formation after the Eastern Mediterranean Transient. One of the drivers for dense water formation is attributed to the decadal reversals of the upper layer Ionian Sea circulation from cyclonic to anticyclonic, which decreases the AW flux into the Levantine Basin, increasing its overall salinity (^43^ and references therein).

From the biogeochemical point of view, the deep Mediterranean is characterized by a low dissolved inorganic carbon/total alkalinity ratio (DIC/TA) and a low Revelle factor, which make it more prone to anthropogenic carbon uptake than any other basin^44,45^. It is therefore important to quantify the rate and distribution of carbon sequestration and transport by the circulation and the biological pump in this region. Moreover, the salinity and temperature of the deep Mediterranean have shown an increasing trend over time^25,46^, which implies significant changes in the water mass formation and thermohaline circulation. These changes need to be documented and understood in terms of their causes and consequences. The winter convection episodes in the WMED have become weaker over time, leading to a decrease of dissolved oxygen concentrations in the intermediate waters^47^. This could affect the carbon export and the mesopelagic marine ecosystems, resulting in new balances within the trophic chain (e.g.^48–54^).

### Cruise summaries

#### TAlPro2016 Cruise

The TAlPro2016 cruise (Palermo, Italy - Barcelona, Spain, RV ANGELES ALVARIÑO, 18.08.2016 - 29.08.2016) occupied three hydrographic transects, one across the relatively flat and deep Algero-Provençal basin, one across the deep and rugged Tyrrhenian Sea, and an additional one across the Balearic Sea (not foreseen in the Med-SHIP design). A total of 43 stations were realised (grey circles in Fig. 1), where physical (temperature, salinity, pressure, velocity) and biogeochemical parameters (oxygen, nutrients, carbonate system) and transient tracers (CFCs and SF_6_) were measured.

The main focus of the cruise was the north-south transects across the WMED, between its northern and southern shores. Along the different sections, CTD stations including sampling of chemical parameters were conducted approximately every 10–30 nm. In addition, underway thermosalinograph and vessel-mounted ADCP measurements were performed between CTD stations.

The specific objectives of the TAlPro2016 cruise were (i) to observe the state of the Western Mediterranean Transition, (ii) to quantify the ventilation of different parts of the WMED, (iii) to assess the rate of warming and salinification, mainly of the intermediate and deep layer, (iv) to monitor the biogeochemical status of the basin, and (v) to update the quantification the uptake of anthropogenic carbon in the deep basin.

#### CRELEV2016 Cruise

The CRELEV2016 cruise (Piraeus - Piraeus, Greece, RV AEGAEO, 02.06.2016 −10.06.2016) was carried out in the Cretan Sea and in the Cretan Passage, south of Crete. The cruise was restricted within the limits of the Greek exclusive economic zone. In total 29 stations were sampled where CTD multiparameter profiles and samples for dissolved oxygen and nutrients were collected (black circles in Fig. 1), according to the GO-SHIP recommendations. On a subset of stations other samples were collected for biogeochemical (carbonate system, dissolved and particulate carbon, nitrogen and phosphorus) and biological (chlorophyll, plankton abundance and biomass, pigment analysis, others) parameters.

The main objective of the CRELEV2016 cruise was to examine a comprehensive set of parameters in the EMED in order to quantify variability and trends of physical and biogeochemical properties. The specific objectives of the cruise were (i) to observe the present post-convective state of the Cretan Sea, in relation to the state of the upper layer Ionian circulation, (ii) to observe the present state and to quantify changes of the hydrographic and biogeochemical properties of the Cretan Sea, the Cretan Strait and the Levantine Basin, (iii) to quantify the uptake of anthropogenic carbon in the EMED, (iv) to quantify changes in water formation rates and circulation in the EMED.

#### ESAW Cruises

The ESAW cruises (Split - Split, Croatia, RV BIOS DVA, 10.12.2015 −15.12.2015 and 05.04.2016 −10.04.2016) were conducted to obtain the multidisciplinary characterization of the Adriatic waters before and after the wintertime conditions, i.e., December 2015 and April 2016, hereinafter referred to as ESAW2015 and ESAW2016. The cruises were carried out in the middle and southern Adriatic Sea.

A detailed dataset was collected to compare physical and biogeochemical characteristics during pre- and post-wintertime conditions. The Adriatic Sea is characterised by a high variability due to the influence of local climate and meteorological conditions, as well as due to the remote effects of the lateral water exchange within the Adriatic and between the Adriatic and Ionian seas. Data were collected along two shore-to-shore key transects (Gargano-Split and Bari-Dubrovnik) and in the mid Adriatic depressions. A total of 55 hydrographic stations were completed (24 during ESAW2015 and 31 during ESAW2016, see red + orange + yellow circles in Fig. 1). The scientific interest was focused on (i) the regeneration processes occurring in the deep waters in the middle and southern Adriatic pits, and (ii) on the distribution of the EIW in the Adriatic Sea, which has an important role in preconditioning the dense water formation, and in imprinting biogeochemical and biological processes.

### Data provenance

- CTD multiparametric probe and underway acquisitions.
- Discrete samples.

## Methods

### Conductivity-temperature-depth (CTD) measurements

#### CTD setup and configuration

During all cruises, the CTD-rosette systems were equipped with a sonar altimeter to reach the deepest point at each station maintaining a safe distance from the bottom of about 5–10 m, depending on the sea conditions. The CTDs were left soaking at the surface for a few minutes before the beginning of the downcast in order for the water pump to activate and until the conductivity cells were stabilized.

Details on Sea-Bird SBE911 + CTD sensor configurations used during the TAlPro2016 cruise are shown in the cruise report^20^. The 24-bottles rosette system SBE32 was equipped with 12 litres Niskin bottles. The rosette was coupled with an SBE43 oxygen sensor, as well as with WETLabs Fluorometer and Turbidimeter.

During the CRELEV2016 cruise a Sea-Bird SBE911 + CTD system attached to a SBE32 carousel water sampler was used, equipped with 12 Niskin bottles (12 litres capacity). An SBE43 oxygen sensor was also used, as well as a Chelsea AquaTracka III fluorometer and a Chelsea AlphaTracka Mk II transmissometer.

During the ESAW cruises the CTD used was a Sea-Bird SBE911 + , coupled with an SBE43 oxygen sensor, a WETLabs FLNTURTD sensor for fluorescence and turbidity, and a WET Labs C-Star transmissometer. The CTD was attached to a rosette sampler holding 12 Niskin bottles (8 litres capacity) for water sampling. A few additional CTD casts were conducted using a separate self-recording SBE25 probe (pressure, temperature and conductivity sensors), sampling at 8 Hz.

#### CTD operations and data processing

For all cruises, the vertical profiles of all parameters were obtained by sampling the signals at 24 Hz, with the CTD/rosette going down at a speed of about 1 m/s. The data were transmitted on-line to the ships’ computer and viewed in real time using *Sea-Bird SeaSave* software (https://www.seabird.com/asset-get.download.jsa?id=54627862734). Data were pre-processed on board using *SBE Data Processing* software (https://www.seabird.com/asset-get.download.jsa?id=54627862733) in the following steps according to the CTD manufacturer’s recommendations: data conversion, low-pass filtering of pressure readings, temporal alignment of CTD’s oxygen sensor, cell thermal mass correction, computing of derived variables, bin averaging at 1 dbar bins and splitting of downcast and upcast. Derived parameters, such as the potential temperature and potential density anomaly referred to 0 dbar, were calculated from original *in situ* data using the TEOS-10 Gibbs function approach^55^. Details on CTD calibration and corrections can be found in Section “Technical Validation”.

### Lowered acoustic doppler current profiler

**Fig. 2:**
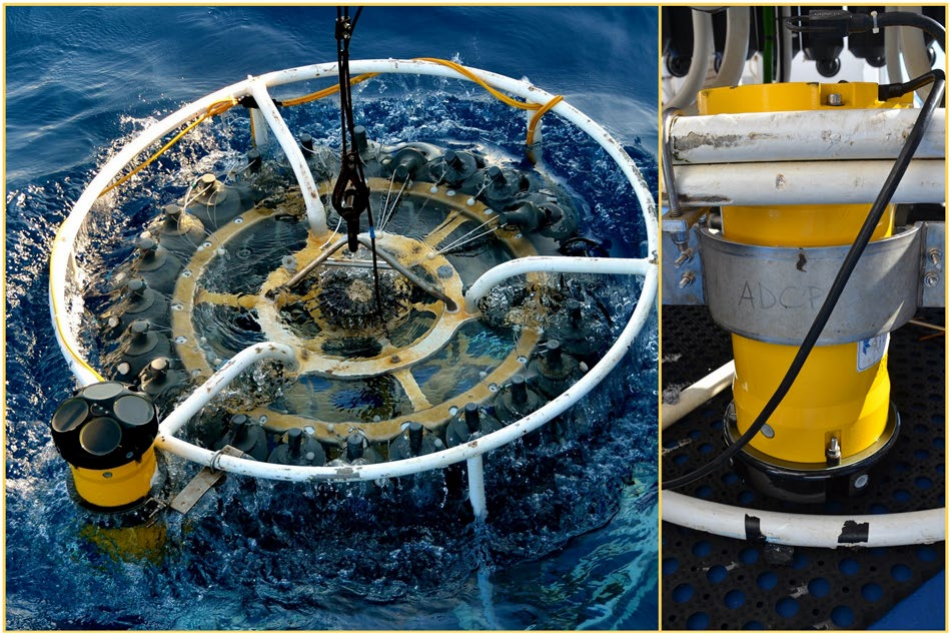
Slave (upward looking, left picture) and master (downward looking, right picture) LADCP set up on the rosette during the TAlPro2016 cruise.

The model RDI Workhorse 300 kHz Lowered Acoustic Doppler Current Profiler (LADCP) was used along with CTD casts to measure current velocity and magnitude. During the TAlPro2016 cruise, two LADCP (a master facing down and a slave facing up, see Fig. 2) were used, while during the CRELEV2016 cruise a single downward-looking LADCP was used. For data post-processing the LDEO LADCP (version 10.16 and version IX, respectively for TAlPro2016 and CRELEV2016) software^56^ was used, which converts the raw LADCP data, combines them with processed CTD data and navigational data, to give the u- and v- velocities profiles from surface to bottom. No LADCP data were collected during the ESAW cruises.

### Discrete water sample analyses

#### Salinity (measurement and calibration)

Water samples were taken at most stations to measure conductivity/salinity and to identify any bias or drift in the conductivity sensors. Standard sample procedure was used^57^. Conductivity was measured with a Guildline Autosal 8400B (TAlPro2022 and ESAW cruises) and 8410 A (CRELEV2016). Once collected, the samples were brought into the room hosting the salinometer to give them time to adjust to the room temperature. The temperature of the water in which the conductivity cell is bathed must be as close as possible to the room temperature and needs to be around 21 °C for optimal results.

The instrument is standardized at the beginning and the end of a set of measurements with Standard IAPSO Seawater. For each sample, three readings of the conductivity ratio were taken (or as many samples as needed to obtain sufficient stability). Salinometer and CTD salinities were compared, new calibration coefficients were computed, and applied to the CTD sensor-derived raw values to obtain corrected salinity values.

#### Dissolved oxygen (measurement and calibration)

Dissolved oxygen (DO) concentrations were measured at all CTD casts with a SBE43 sensor (Sea-Bird Clark electrode polarographic sensor), which has an accuracy of 2% DO saturation (around 5 µmol kg^−1^ at 13 °C with salinity 38). To adjust the sensor response from the drift and offset, seawater samples were collected at almost all stations from surface to deep waters for discrete Winkler analysis performed onboard.

Winkler-titrated water samples were used as the DO reference concentrations^57^. Sampling was done with the recommended precautions to prevent any biological activity and gas exchanges with the atmosphere^58^. Temperature of the water in the sampling bottle was measured during sampling with a digital thermometer. Samples were then immediately fixed after collection with the reagents (#1: manganous chloride and #2: sodium hydroxide – sodium iodine) added with semi-automated dispensers according to^59,60^. The bottles were shaken for about 1 minute to bring each molecule of dissolved oxygen into contact with manganese (II) hydroxide. After fixation the precipitate was allowed to settle below the half of the bottle (min. 4 hours). The bottles were kept in the dark. Before titration, the precipitated hydroxides were dissolved with sulfuric acid. During TAlPro2016 titration was performed directly in the sample bottle, while during CRELEV2016 the solution was transferred carefully into a titration beaker. This was done because of technical problems with the titration equipment, which prevented us from performing the titration in the sample bottle. The titration was carried out with a standardized thiosulphate solution using a Dosimat Metrohm (for TAlPro2016 and CRELEV2016). The thiosulfate solution was calibrated by titrating it against a potassium iodate certified standard solution of 0.0100 N (CSK standard solution WAKO).

During ESAW cruises, water samples for DO determination were collected in 50 mL calibrated BOD (Biological Oxygen Demand) bottles and concentrations were measured by the Winkler iodometric titration using a Mettler-DL21 titrator for the determination of the equivalence point^61^. Temperature of the water in the sampling bottle was measured during sampling with a GMH 3475 digital thermometer. The precision of the measurements evaluated on five determinations on subsamples collected from the same Niskin bottle was 0.1%. After the cruise, these data have been used to check the sensor’s response. After a thorough inspection and comparison, the SBE43 sensor data were retained good enough and were kept without change.

#### Dissolved inorganic and organic nutrients

During TAlPro2016, samples for dissolved inorganic nutrients were collected at all stations from surface to deep waters (from 9 to 24 depth levels each cast). These unfiltered samples were collected through 60 mL polyethylene flasks, poisoned with 100 μl HgCl_2_ (6 g/L for 100 µl of solution for 20 ml of samples, i.e. 20 μg L^−1^) and stored in a dark room at 5 °C until analysis^62^. In previous works, comparisons have been made between freezing and poisoning, with no significant differences found (SOMLIT Intercomparisons, Service d’Observation en Milieu Littoral, https://www.somlit.fr/en/intercomparisons/). Samples can be stored for up to a year in the dark at room temperature^62^. All nutrient samples were analysed by a standard automated colorimetric system, using a Seal Analytical continuous flow AutoAnalyser III (AA3) at IMEV (Institut de la Mer de Villefranche, Sorbonne University & CNRS). Nitrate (NO_3_^−^) ions were analysed according to the protocol^63^ with a detection limit of 0.01 μmol L^−1^. Phosphate (PO_4_^3−^) ions were analysed according to the protocol^64^ with a detection limit of 0.02 μmol L^−1^. Silicate (Si(OH)_4_) ions were analysed according to the protocol^58^ with a detection limit of 0.02 μmol L^−1^.

During CRELEV2016 nutrients were sampled at 29 stations at all the depths of closure of the Niskin bottles. Overall, 561 unfiltered samples were collected. Two subsamples were collected for each sampling depth. For the onboard analyses, seawater was collected in acid washed polycarbonate 10 mL vials and preserved at 4 °C until analysis, another subsample was collected in acid washed 20 mL HDPE vial and was immediately stored at −20 °C. The determination for nitrate, nitrite, phosphate and silicate were performed onboard by a colorimetric four-channel Continuous Flow Analyzer QuAAtro Seal Analytical following the methods reported in^65^. In addition, during CRELEV2016 cruise, the MAGIC25 procedure^66^ was followed for the determination of low phosphate concentrations. In the ultra-oligotrophic euphotic zone of the EMED the determination of phosphate with the standard phosphomolybdic blue method is difficult, because phosphate concentrations are typically at or below the detection limit of the standard method^58^. MAGIC25 procedure was used instead of the classic phosphomolybdic blue method when the phosphate concentration was below 200 nM, in the layer 0–200 m, at all 29 stations. According to the MAGIC25 method, phosphates were pre-concentrated by 25 times, thus the procedure allowed detection of nanomolar concentration in seawater (detection limit ~1 nM). In total MAGIC25 measurements were performed at 263 samples in duplicates, for a total of 526 phosphate analyses on board. For the determination of phosphates with MAGIC25 procedure, 250 mL of seawater was sampled in duplicates from the Niskin bottles at high density polycarbonate bottles of 250 mL. Turbidity blanks in duplicates were determined at several stations and depths. The bottles were then centrifuged with NaOH. The final volume of the MAGIC25 concentrate was 10 mL, which corresponded to a 25 times preconcentration. The colorimetric procedure was finally performed on board using a PERKIN-ELMER 25 Lambda spectrophotometer. Three calibration curves were performed on board, in duplicates, in seawater taken from the sampling area at 50 m depth and were used for the calculation of the phosphate concentration in the seawater samples. Moreover, during CRELEV2016 a total of 276 samples for total dissolved nitrogen and phosphorus were collected at 16 stations at standard depths. Samples for the analyses of Total Dissolved Nitrogen (TDN) and Phosphorus (TDP) were filtered on 0.2 μm filters in a Nalgene polysulfone 0.5 litres filtration system under mild vacuum and collected in 125 mL HDPE bottles from the Niskin bottle. The filtrate was collected in acid washed polyethylene vials rinsed with filtered seawater and immediately frozen (−20 °C) until laboratory analysis. The analysis was performed by wet persulfate oxidation, according to^65^. Dissolved organic nitrogen (DON) and phosphorus (DOP) have been computed as follows: DON = TDN – (N-NO_2_ + N-NO_3_) and DOP = TDP – P-PO_4_.

During ESAW cruises samples for nitrate, nitrite, ammonium, phosphate and silicate analysis were collected with prefiltration on GF/F (glass fiber) Whatman filters in polyethylene 100 mL capacity vials and were immediately stored at −20 °C. The analyses have been performed in the onshore National Institute of Oceanography and Applied Geophysics (OGS, Italy) laboratory by a colorimetric four-channel continuous Flow QuAAtro Seal Analyzer following the methods reported in^65^. Dissolved organic nitrogen (DON) and phosphorus (DOP) have been computed as follows: DON = TDN – (NO_2_^−^ + NO_3_^−^ + NH_4_^+^) and DOP = TDP – PO_4_^3−^.

#### CO_2_ system variables

During the TAlPro2016 cruise four CO_2_ variables were measured: pH, Total Alkalinity (TA), Dissolved Inorganic Carbon (DIC) and carbonate ion concentration (CO_3_^2−^). During CRELEV2015 three CO_2_ variables were measured (pH, TA, DIC) and during ESAW cruises two variables (pH and TA) were measured. A different subsection is devoted for each cruise to take into account some differences in the methodologies, although there are overlaps in the methods that were used.

#### TAlPro2016 cruise

Due to on board time limitations, a complete sampling scheme for all stations and depths was unachievable: no CO_2_ data was sampled in the Balearic Sea, while in the other two sections (Fig. 1) DIC, pH, TA, and CO_3_^2−^ covered respectively 55%, 90%, 31% and 34% of the total discrete depths sampled. In the following sections a brief description of the methods and quality control schemes for the different CO_2_ variables is given, while more information can be found in the TAlPro2016 cruise report^20^.

##### DIC determination

DIC samples were analysed on board with a VINDTA (Versatile INstrument for the Determination of Total inorganic carbon and titration Alkalinity) 3D system (www.marianda.com) coupled to a UIC 5011 coulometer, briefly, seawater CO_2_ was extracted adding phosphoric acid within a stripping chamber, the generated CO_2_ was carried by a pure N_2_ gas stream into a coulometer cell where the coulometric titration was performed^67^. Samples for DIC were collected after transient tracers and dissolved oxygen, preferably in 500 mL borosilicate bottles, alternatively in 200 mL topaz bottles. Bottles were rinsed and filled smoothly from the bottom till overflowing, leaving a headspace of 1% the bottle volume. Samples were stored at room temperature in the dark until analysis, maximum 3 days after sampling.

##### pH determination

Spectrophotometric pH in seawater was measured following^68^ using a manual approach. Samples were collected in cylindrical optical glass 10-cm pathlength cells, which were filled to overflowing and immediately stopped. All the absorbance measurements were obtained in the thermostated (25 ± 0.2 °C) cell compartment of a BECKMAN DU800 single beam spectrophotometer. After blanking with the sampled seawater without dye, 50 μL of the unpurified m-cresol purple (2 mM, Sigma Aldrich) solution was added to each sample. The absorbance was measured at four fixed wavelengths (434, 487.6, 578 and 730 nm). pH in the total hydrogen ion concentration scale, is calculated using the formula by^68^ and reported at 25 °C, hereinafter pH25T. The injection of the indicator slightly perturbs the sample pH, the double addition correction was applied. The magnitude of this correction over our pH range is small, ranging from 0.004 to 0.005 pH units, which are added to the uncorrected pH values.

##### TA determination

TA was analysed following the double end point potentiometric technique by^69^, further improved by^70^. This technique is faster than the whole curve titration, but comparable^71^. Seawater samples for TA were collected after pH samples in 600 mL borosilicate bottles, filled to overflowing and immediately stopped. Samples were stored in the dark at laboratory temperature until analysis, usually no later than 2 days. The TA potentiometric titrations were done with an automatic potentiometric titrator combined with a glass electrode and a Pt-1000 temperature probe. The system was coupled with a 5 mL exchangeable unit filled with 0.1 N hydrochloric acid to a final pH of 4.40^69^. The electrode was standardised with a 4.41 pH phthalate buffer made in CO_2_ free seawater^70^. Salinity variations after the titration were considered in the final TA calculation. Each sample was analysed twice and the reported values are means of two values.

##### CO_3_^2−^ determination

The carbonate ion concentration was determined manually following the spectrophotometric method as in^72^ with the modifications proposed by^73^. CO_3_^2−^ samples were collected after TA in cylindrical optical quartz 10-cm pathlength cuvettes, filled to overflowing and immediately stopped. After sampling the cells are immediately stabilised at 25 °C using the same temperature control as for pH. All the absorbance measurements were obtained in the thermostated (25 ± 0.2 °C) cell compartment of a BECKMAN DU800 single beam spectrophotometer. After blanking with the sampled seawater without dye, 20 μL of the titrant Pb(ClO_4_)_2_ solution (0.022 M, Fisher Scientific, 99.99% purity dissolved in distilled water) was added to each sample. The absorbance was measured at three wavelengths (234, 250 and 350 nm). The final CO_3_^2−^ concentration at 25 °C was calculated with the^73^ formula which includes a titrant induced perturbation term.

#### CRELEV2016 cruise

##### DIC determination

Seawater samples for the determination DIC were drawn into 500–1000 mL borosilicate glass bottles sealed with glass stoppers according to the collection methods of^74^. The samples were poisoned with saturated mercuric chloride solution to prevent further biological activity, stored at room temperature away from light and were analysed onshore within 6 months. Totally, 184 samples from 13 stations were collected for the determination of DIC. Samples were analysed by a VINDTA 3 C system based on a coulometric procedure using 10% phosphoric acid and a continuous nitrogen carrier gas supply with adjusted pressure to 1.5 bar, in a closed cell. The sea water sample (20 mL) is acidified with phosphoric acid (10% in 0.7 M NaCl), which converts all carbonate species into CO_2_ gas. The generated CO_2_ is carried into the coulometric cell using an inert gas (N_2_), and titrated coulometrically using internally prepared anode and cathode solutions. The sampling, titration and DIC calculation of the sea water samples is controlled by LabVIEW TM software (https://www.ni.com/it-it/shop/labview.html). All samples were measured at 25 °C with a temperature-controlled water-bath. As a guidance for the laboratory work and preparation of internal standards and other material, the operating manual for the VINDTA 3 C by^75^ was used.

##### pH determination

Samples were collected directly in the spectrophotometric cylindrical cells with 10 cm path length by rinsing twice and filling by overflowing twice the cell volume and stoppering with Teflon caps. All pH spectrophotometric measurements were performed onboard within 3 hours from subsampling. The samples of station 3 and station 5 were maintained and measured at 20 °C, while samples of stations 7, 9, 11, 13 and 15 were maintained and measured at 18 °C, closer to *in situ* temperature. But, because of condensation on the cell walls and of thermoregulation problems, from station 18 the samples were maintained and measured at 25 °C, to be closer to ambient temperature. Overall, 247 pH measurements were performed onboard. Samples were collected at standard depths at 16 stations. Samples were measured using a single beam spectrophotometer (Cary 50 Scan UV-Visible) with a thermostatic cell holder and a purified indicator dye m-cresol purple^76^ following the Standard Operating Procedure (SOP6b^74^). The temperature in the cell holder was maintained constant by a cryothermostat Lauda ECO Silver and the samples were pre-equilibrated at established temperature in a thermostat Pol-ECO thermostat.

##### TA determination

Samples were collected into 250 mL narrow-necked borosilicate bottles filling twice the volume from the bottom. Each bottle was poisoned with 100 µL of saturated mercuric chloride (HgCl_2_), to halt biological activity, and immediately stored at 4 °C in the dark until analysis. Total alkalinity was determined within 6 months in an onshore OGS laboratory by potentiometric titration in an open cell (SOP 3b^74^) on 35 samples collected at stations 23, 25, 27 and 28. TA analyses were also performed on the samples collected at the other stations by VINDTA3C instrument at HCMR laboratory. This instrument, allows the determination of TA using an open cell potentiometric acid titration method^74^, however, the results from these analyses were discarded due to technical issues encountered at the electrode in the alkalinity cell, therefore only TA values measured on a 35 samples at OGS laboratories by open cell titration were kept.

#### ESAW cruises

##### pH determination

The pH was measured using a single-beam spectrophotometer (Cary 50 Scan UV-Visible) with a thermostated cell holder and a purified indicator dye m-cresol purple following the SOP3b^74^. The temperature in the cell holder was maintained at 14.0 °C by a cryothermostat Lauda ECO Silver. To avoid CO_2_ gain or loss, unfiltered seawater samples were collected directly into quartz cuvettes with 10 cm pathlength, leaving no head space and pre-equilibrated at 14.0 °C in a Pol-ECO thermostat. pH was always measured within 1–3 hours from subsampling.

##### TA determination

For the total alkalinity, samples were pre-filtered on glass-fibre filters (Whatman GF/F) into 250 mL narrow-necked PVC bottles. Filtration was performed to remove phytoplankton cells and particles of CaCO_3_, derived from calcifying organisms, which respectively can interact with HCl titrant solution and dissolve during measurements due to acid additions, inducing a relevant error in the exact estimation of the TA concentration^77^. Each bottle was poisoned with 100 μL of saturated mercuric chloride (HgCl_2_) to halt biological activity and stored at 4 °C in the dark until analysis. TA was determined within 6 months in an onshore OGS laboratory by potentiometric titration in an open cell^74^.

#### Dichlorodifluoromethane and sulfur hexafluoride

Measurements of the transient tracers dichlorodifluoromethane (CFC-12 or freon-12) and sulfur hexafluoride (SF6) are used to characterize ventilation in the Mediterranean, and particularly temporal changes in ventilation (e.g.^44,78,79^. During the TAlPro2016 and CRELEV2016 cruises, the gas chromatograph/purge and trap (GC/PT) systems were used for the measurements of the transient tracers CFC-12 and SF6. The systems are modified versions of the set-up normally used for the analysis of CFCs^80,81^. Samples were collected in 250 mL ground glass syringes. An aliquot of about 200 mL of the samples was injected into the analytical systems. The analytes were stripped out of the water phase by a flow (120 mL /min) of ultra-clean N2 during 5–6 minutes to the trap. The trap consists of 100 cm of 1/16” tubing packed with 70 cm Heysep D and is kept at −60 °C during the trapping phase. The trap was desorbed at 120 °C and the analytes passed on to the GC. The GC was setup with a 1/8” main column packed with 180 cm Carbograph 1AC (60–80 mesh) and a 20 cm Molsieve 5 A postcolumn, kept isothermal at 50 °C. The pre-column was packed with 10 cm Porasil C and 20 cm Molsieve 5 A in a 1/8” stainless steel column but had to be shortened to 15 cm Porasil C and 20 cm Molsieve 5 A. Detection was performed on an Electron Capture Detector (ECD). This set-up allowed efficient analysis of SF6 and CFC-12.

During the ESAW cruises water samples for the determination of CFC-12 and SF_6_ concentrations were collected from the Niskin bottles using 300 mL glass ampules. The ampules were directly attached to the Niskin bottles with a stainless-steel tubing system to prevent contact with the atmosphere during the sampling process (e.g.^82^). The ampules were flushed with 3 times the volume during sampling. The samples were cooled in a water bath at ~10 °C to prevent outgassing of the trace gases before they were flame sealed and stored in aluminium boxes during the cruise; flame sealing was always performed immediately after sampling. The measurements of the samples were conducted onshore in the tracer lab at GEOMAR Helmholtz Centre for Ocean Research Kiel within one year since sampling. The two transient tracers were simultaneously analysed using a purge and trap GC-ECD system as described in^83^.

#### Additional variables

A number of additional variables, not strictly included in the Med-SHIP/GO-SHIP sampling strategy, which are not further discussed here, have been collected: nitrogen and oxygen isotopes during TAlPro2016 and particulate organic carbon (POC), particular nitrogen (PN), carbohydrates (total, mono- and poly-saccharides), and chlorophyll-a biomass during CRELEV2016, chlorophyll-a biomass, phytoplankton population structure (microplankton and nanoplankton), microbiology sampling, zooplankton sampling and benthos sampling during ESAW. Only the additional variables of the CRELEV2016 cruise are included in the dataset submitted to the repository listed in Table 3.

### Underway measurements

#### Thermosalinograph

**Fig. 3:**
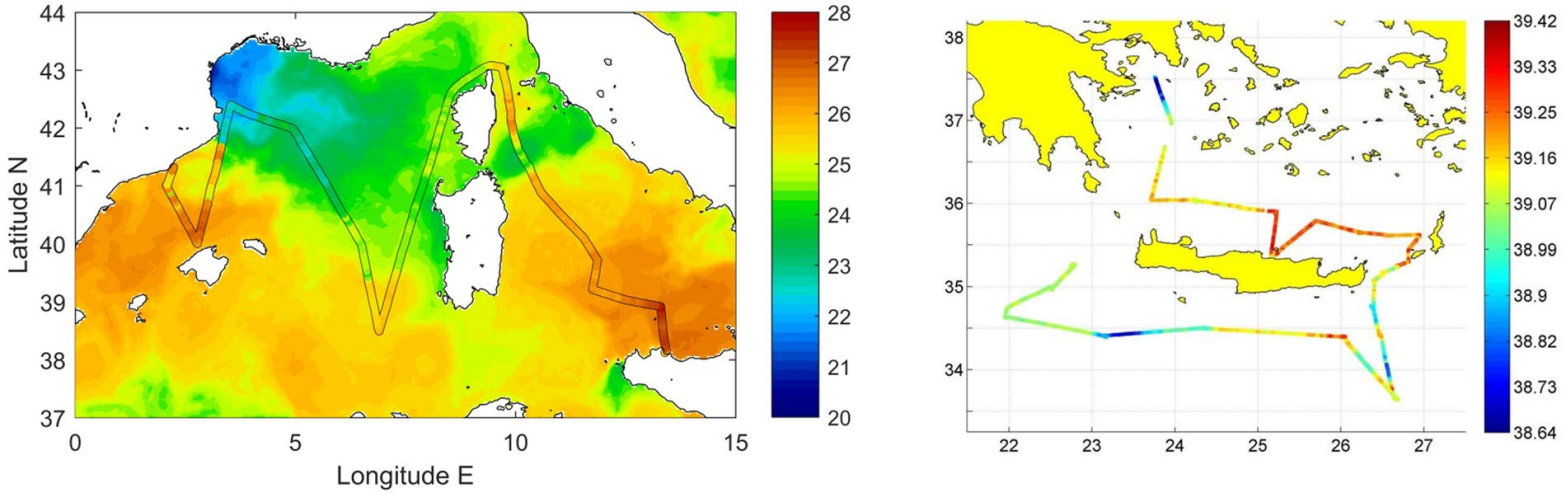
(left) Sea Surface Temperature from Copernicus Marine Environment Service (CMEMS) reanalysis (August 2016) and temperature measured by the thermosalinograph during TAlPro2016; (right) salinity measured by the thermosalinograph during CRELEV2016.

During TAlPro2016 and CRELEV2016 a thermosalinograph collected underway temperature and salinity data (plus fluorescence for TAlPro2016) along the cruise routes (Fig. 3). No thermosalinograph data were collected during the ESAW cruises. An SBE21 thermosalinograph was used during CRELEV2016, while during TAlPro2016 the thermosalinograph system consisted of a SBE21 together with a SBE38 thermometer. Both systems worked independent from each other throughout the cruise. While temperature is taken at the water inlet in about 5 m depth (SBE38), salinity is estimated within the interior thermosalinograph from conductivity and interior temperature (SBE21). A Turner fluorometer (10-Au-005) was connected to the system.

#### Vessel-mounted acoustic doppler current profiler

**Fig. 4:**
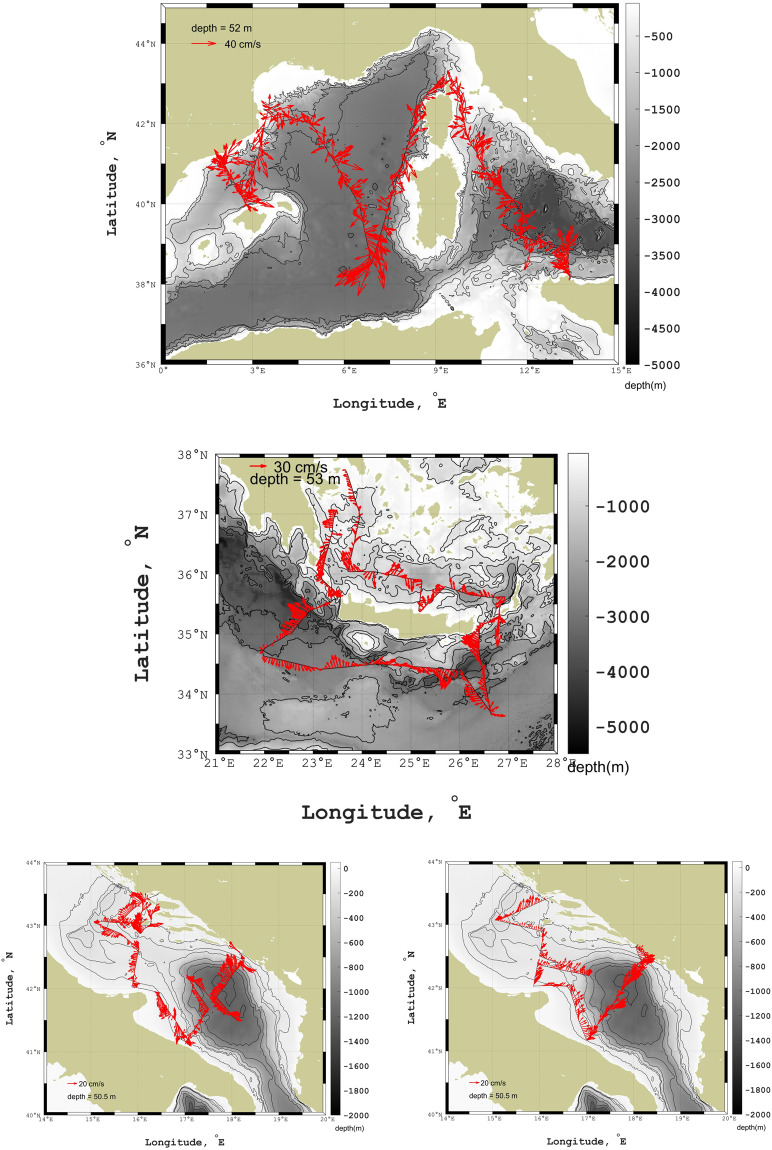
Currents measured during TAlPro2016 at 52 m depth (upper panel), during CREVLEV2016 at 53 m depth (middle panel), during ESAW2015 (bottom left panel) and ESAW2016 (bottom right panel) at 50.5 m depth.

The hydrographic data set comprises current measurements by hull-mounted ADCPs (Fig. 4). During TAlPro2016 a RDI Ocean Surveyor (150 KHz) has worked along the ship’s route. The depth range of the current profiler is about 300 m (number of cells was set to 60, bin size to 8 m). During CRELEV2016 a RDI Ocean Surveyor (75 KHz) was used to monitor ocean currents up to depths of 700 m (number of bins was set to 50, bin size to 15 m). Both systems ran in narrowband mode. During the ESAW cruises, a RDI broadband WorkHorse Mariner (300 KHz) registered horizontal velocities with a range of about 90 m (number of cells was set to 50, bin size to 4 m).

All systems were connected to the Global Positioning System (GPS) and controlled by computers using the conventional RDI VMDAS (Vessel-mounted Data Acquisition System, TRDI) software package, installed under a MS Windows system with a pinging set to be as fast as possible. No interferences with other used acoustical instruments were observed.

## Data Records

The datasets described in this paper have been submitted to repositories, ensuring accessibility and long-term preservation, as detailed in Table 3. The data are available at the following repositories: PANGAEA (^83–87^), NCEI/NOAA (^88,89^ and NODC-OGS (^90–96^).

The details on file formats and types are described in the following sections. It is essential to underline that the primary objective of this paper is to offer a comprehensive depiction of cruises conducted under the Med-SHIP initiative. These cruises took place on diverse vessels, led by independent teams, each operating autonomously. Consequently, the data records from these cruises exhibit a degree of heterogeneity and inconsistencies, evident in variations such as dataset naming conventions and repositories used. Through this paper, we underscore the imperative for improved coordination in forthcoming basin-wide efforts within the Med-SHIP framework. This coordination should aim for a high degree of consistency in the provision and description of collected data.

### Physics data of TAlPro2016 cruise

This is a tab-delimited dataset containing the data description^83^, the events (station listing and details on coordinates and time), the parameter list and corresponding columns (depth, pressure, temperature from 2 sensors, conductivity from 2 sensors, dissolved oxygen, turbidity, fluorescence, salinity from 2 sensors, density, sigma-theta, potential temperature, absolute salinity, conservative temperature) and the licence.

### Chemistry data of TAlPro2016 cruise

This is a tab-delimited dataset containing the data description^84^, the events (station listing and details on coordinates and time), the parameter list and corresponding columns (pressure, salinity, temperature, sulfur hexafluoride, conductivity, freon-12, dissolved oxygen from sample, dissolved oxygen from CTD, nitrite, nitrate, phosphate, silicate, dissolved inorganic carbon, total alkalinity, pH, carbonate ion, turbidity, fluorescence, salinity, density, sigma-theta, potential temperature, absolute salinity, conservative temperature), parameter flags and the licence.

### Lowered acoustic doppler current profiler data of TAlPro2016 cruise

This is a tab-delimited dataset containing the data description^85^, the events (station listing and details on coordinates and time), the parameter list and corresponding columns (depth, east-west current velocity, north-south current velocity, vertical current velocity, elapsed time) and the licence.

### Vessel-mounted acoustic doppler current profiler data of TAlPro2016 cruise

This is a netCDF file with an extensive header describing metadata^86^, parameters names and units, as well as the processing steps, containing the following parameters: depth, zonal velocity component, meridional velocity component, received signal strength, percent good pings, editing flags, ship heading, ADCP transducer temperature, number of pings averaged per ensemble, ship zonal velocity component, ship meridional velocity component.

### Thermosalinograph data of TAlPro2016 cruise

This is a tab-delimited dataset containing the data description^87^, the events (with details on coordinates and time), the parameter list and corresponding columns (depth, elapsed time, temperature from 2 sensors, salinity from 2 sensors, fluorescence, conductivity from 2 sensors) and the licence.

### Physics data of ESAW cruises

These are comma-separated values (csv) datasets in Ocean Data View (ODV) data format (https://odv.awi.de)^90,93^, containing the cruise details, the events (station details on coordinates and time), the parameter columns (bottom depth, pressure, conductivity from 2 sensors, temperature from 2 sensors, salinity from 2 sensors, dissolved oxygen, fluorescence, turbidity from 2 sensors, distance from the bottom, smoothed dissolved oxygen, smoothed fluorescence, smoothed turbidity).

### Chemistry data of ESAW cruises

These are comma-separated values (csv) datasets containing the cruise details^91,94^, the events (station details on coordinates, time and bottle position), the parameter columns (bottom depth, pressure, depth, temperature, salinity from CTD, salinity from sample, dissolved oxygen from CTD, dissolved oxygen from sample, nitrite, nitrate, phosphate, ammonium, silicate, total dissolved nitrogen, total dissolved phosphorus, total alkalinity, pH on total scale, *in situ* pH, dissolved organic carbon, sulfur hexafluoride, freon-12).

### Vessel-mounted acoustic doppler current profiler data of ESAW cruises

These are comma-separated values (csv) datasets in Ocean Data View (ODV) data format (https://odv.awi.de)^92,95^, containing a header with the cruise and station details, and the following parameters: depth, eastward horizontal velocity, northward horizontal component.

### Physics data of CRELEV2016 cruise

The single compressed file in^96^ contains all cruise files^96^. The physical data are contained in the file named CTD_dataset_CRELEV-2016.txt. This is a text ASCII file dataset in Ocean Data View (ODV) data format (https://odv.awi.de) containing a header with the cruise details, information on each station, and the following parameters: bottom depth, pressure, temperature, dissolved oxygen, fluorescence, turbidity, potential temperature, salinity, potential density anomaly, corrected dissolved oxygen.

### Chemistry data of CRELEV2016 cruise

The single compressed file in^96^ also contains the chemical data, in the file named CRELEV-2016_bottle.txt^96^. This is a text ASCII file dataset in Ocean Data View (ODV) data format (https://odv.awi.de) containing a header with the cruise details, information on each station, and the following parameters: bottom depth, pressure, temperature, salinity, turbidity, fluorescence, chlorophyll, dissolved oxygen from CTD, dissolved oxygen from samples, nitrite, nitrite + nitrate, nitrate, phosphate, phosphate with MAGIC procedure, silicate, total dissolved nitrogen, total dissolved phosphate, dissolved inorganic carbon, total alkalinity, *in situ* pH, freon-12, sulfur hexafluoride, dissolved organic carbon, particulate nitrogen, particulate organic carbon, monosaccharides, total saccharides, polysaccharides.

### Lowered acoustic doppler current profiler data of CRELEV2016 cruise

The single compressed file in^96^ also contains the LADCP data, in a folder named ADCP-2016^96^, and this folder contains one file for each LADCP/CTD station (station_name.txt). These are text ASCII files containing a header with the station details and the following columns: depth, east-west current velocity, north-south current velocity, error velocity.

### Vessel-mounted Acoustic Doppler Current Profiler data of CRELEV2016 cruise

The single compressed file in^96^ also contains the VM-ADCP data^96^, in a folder named ADCP-route, and this folder contains the whole route data acquisition divided in 31 files (route#.txt) of different lengths. These files are text ASCII files that are organized as a matrix where the first 3 rows contain details on time and coordinates and below there are the following rows (corresponding to each time step and lon/lat pair): depth, east-west current velocity, north-south current velocity.

### Thermosalinograph data of CRELEV2016 cruise

The single compressed file in^96^ also contains the thermosalinograph data^96^, in the file named CRELEV-2016_Thermosalinograph.txt. This is a text ASCII file dataset containing details on time and coordinates as well as the following parameters: temperature, salinity

## Technical Validation

To ensure the quality in terms of precision and accuracy of the collected data, we performed several checks and validations on the data before processing and analysing them. In particular, we checked and quality controlled the chemistry data from Niskin bottles using various methods. In general accuracy is ensured by following best practices recommendations^57^. The different methods are described for each measurement in the following paragraphs.

### Temperature and salinity data

For all cruises, we followed the best practices procedures^57^ and recommendations from the CTD manufacturer. Conductivity sensors have been recently calibrated: for TalPro2016 cruise all sensors mounted on the Sea-Bird SBE911 + CTD system were calibrated in May 2016 at the manufacturer’s; for ESAW cruises, pre- and post-cruise calibration have been performed at the Centre for Oceanographic Calibration and Metrology at OGS; for CRELEV2016 cruise the sensors were calibrated between December 2015 and January 2016 at the Sea-Bird facility in Germany (temperature and conductivity) and in the US (oxygen).

The CTD model used during all cruises (i.e. Sea-Bird Electronics, Inc. SBE911 + plus) has the following initial accuracies: conductivity ± 0.0003 S/m, temperature ± 0.001 °C, pressure ± 0.015% of full scale range.

Salinity data were corrected after the cruises using the conductivity measurements obtained from water samples and analysed with a salinometer or performing a laboratory sensor calibration (see subsection “Salinity (measurement and calibration)”). Accuracy for salinometer data is of the order of 0.002 in salinity (precision 0.0001).

**Fig. 5:**
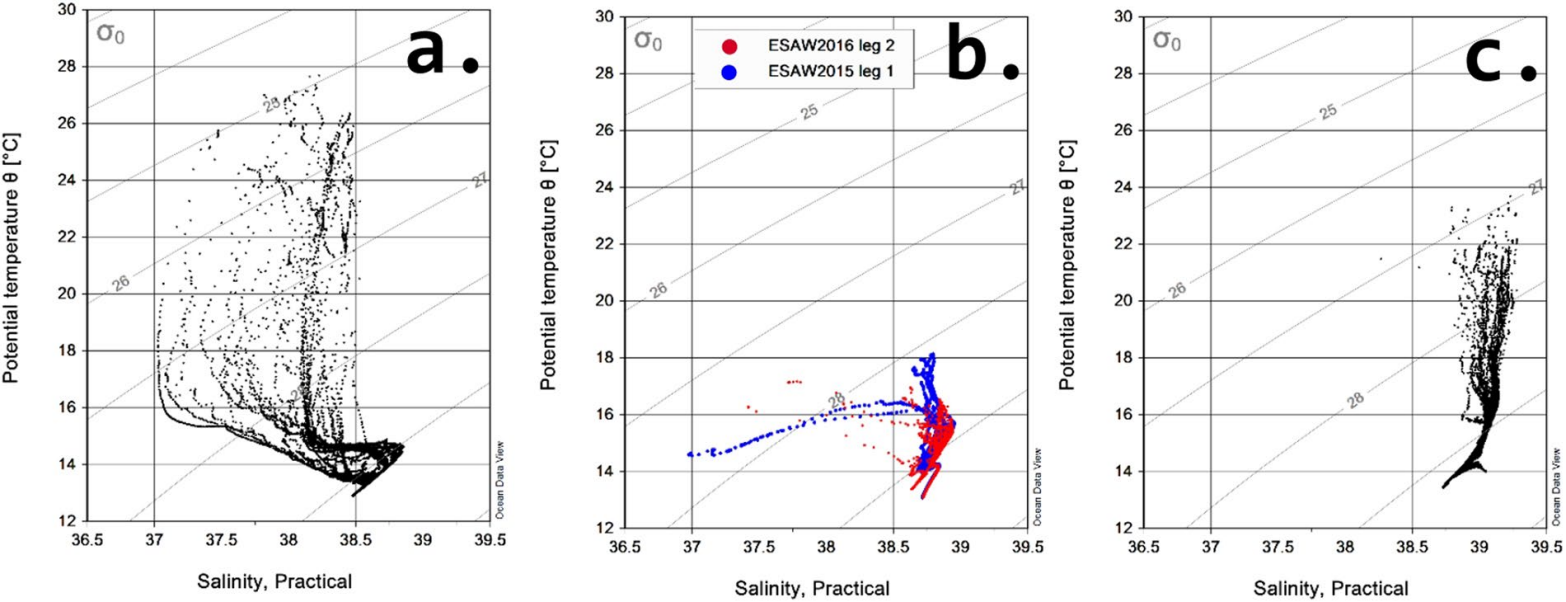
Scatter plot of potential temperature and salinity in the WMED (**a**), in the Adriatic Sea (**b**) and in the EMED (**c**). Grey contour lines indicate potential density anomaly isolines (kg m^−3^).

We checked for any inconsistencies or anomalies in the thermohaline data using potential temperature vs. salinity diagrams (Fig. 5), which describe the wide range of temperature and salinity values found in the upper and deep layers within the Mediterranean basins. We identified and removed any outliers or spurious data points that deviated from the expected potential temperature vs. salinity relationships.

Salinity in the upper layers of the WMED and of the Adriatic ranges between 37 and 38.5. It is distinctively lower than in the EMED, where surface values are higher (around 39). The saltiest intermediate water is the EIW, its properties decrease in temperature and salinity, from 16 °C and >39 in the EMED, 15.5 °C and 38.9 in the Adriatic, to about 15 °C and 38.8 in the WMED. In the Adriatic Sea a patch of salty but cooler water was observed below the EIW. The deep layers in the three basins are distinguished according to their local potential temperature vs. salinity properties, subject both to advective and local convective processes, related to the dense water formation. Generally, the coolest and freshest are deep WMED waters, and warmest and saltiest, those in the EMED. In the latter, the deep waters of the Cretan Sea are saltier and warmer than those in the Cretan Passage.

### Lowered acoustic doppler current profiler data

We checked and calibrated the LADCP data following the recommendations by^56^. To maximize LADCP profile accuracy, depth and sound-speed data, which are usually derived from pressure, temperature and salinity measurements, were used during the post-processing. While some ADCPs are equipped with ancillary sensors for measuring pressure and temperature, LADCP systems more typically use data from high-quality CTDs for this purpose. We also used precision navigation from the shipboard GPS receiver, which is crucial for determining the ship’s drift during the LADCP cast. The ship drift provides a valuable velocity-referencing constraint, which requires that the deployment/recovery positions are known as precisely as possible. Accuracy of LADCP data is typically between 3 and 4 cm s^−1^, an estimate based on comparisons with other independent velocity measurements, such as shipboard ADCP, moored ADCP, or mechanical current meters. No independent assessment for the data of the cruises presented here has been done.

### Dissolved oxygen data

Dissolved oxygen data from the CTD sensors have been either calibrated or checked after each cruise using the measurements done with the Winkler titration method (see subsection “Dissolved oxygen (measurement and calibration)”), which is a standard technique for measuring dissolved oxygen concentration in seawater samples and which sees the use of solutions that are calibrated against certified standard solutions. The reported precision that can be associated with dissolved oxygen data when following the described procedure is 2.2 μmοl O_2_ kg^−1^. Furthermore, for TAlPro2016, a similar method to the one described by^47^ has been used to calibrate the SBE43 data. We matched the data from the upcast profiles with the Winkler samples based on pressure and minimized the residual of the squared differences between the calibrated and water sample DO data. To do this, we adjusted the calibration coefficients of the SBE43 sensor. These coefficients are the DO signal slope, SOC, the voltage at zero DO signal, Voffset, and the pressure factor correction E (see^47^ for details). To ensure high robustness of the DO data adjustment, the number of samples used to determine each set of coefficients was maximised by using the largest possible number of consecutive profiles (i.e. samples with a constant DO_SBE43 vs. DO_Winkler ratio). The total numer of samples used to calibrate the SBE43 sensors were 270 for TalPro2016 and 560 for CRELEV2016.

### Nutrient data

For nutrients we used segmented flow analysis methods with Auto-Analyzers using standard methods^58,63,64^. Furthermore, for CRELEV2016, the precision of the measurement was evaluated on five subsamples collected from the same Niskin bottle closed at 125 meters depth. The resulting standard deviations for nitrate + nitrite, phosphate and silicate were 0.011, 0.008, 0.023 µM, respectively. The limit of detection (LOD), based on the standard deviation of the response, σ, and slope, s (LOD = 3σ/s) were 0.04, 0.008 and 0.02 µM for nitrate + nitrite, phosphate and silicate, respectively. For ESAW, the detection limits were 0.006, 0.02, 0.03, 0.01 and 0.01 µM for nitrites, nitrates, ammonium, phosphates and silicates, respectively.

### Dissolved inorganic carbon data

For DIC, since no calibration unit was available for the system during TAlPro2016, samples of CO_2_ certified reference material (CRM) from Prof. Dickson (Scripps Institution of Oceanography, San Diego, USA, batch #147) were analysed at the beginning and end of each batch of DIC analysis. The ratio between the CRM DIC nominal and measured value was used as an initial calibration factor, while the CRM DIC measured at the end of the batch was used to correct small temporal drifts. Due to time limitations, no typical precision exercise was done for DIC analysis. Some collected DIC bottles were analysed twice to check the reproducibility. Differences were usually less than 2 μmol kg^−1^. Considering on board temperature problems (lab temperatures were high, and not stable, as described in subsection “Comments on quality control and internal consistency of CO_2_ data”) affecting the conservation of the samples until analysis, and probably also the CO_2_ extraction and coulometric reaction, our estimation of the DIC accuracy is 4 μmol kg^−1^. Also during CRELEV2016, the accuracy of the DIC was assessed by measurements of CRM from Prof. Dickson (Scripps Institution of Oceanography, San Diego, USA). The precision achieved was 2 μmol kg^−1^, and based on the CRMs used, the accuracy was ± 12 μmol kg^−1^.

### pH data

For pH, we used spectrophotometric methods and compared the results with CRM. During TAlPro2016, the pH accuracy was checked measuring replicates from CO_2_ CRM (Prof. Dickson batch #147). Two exercises were done: 10–12 samples were carefully drawn from each CRM bottle to avoid bubbles. The corresponding theoretical pH25T value for this batch using the dissociation constants from^97,98^ is 7.9197. The measured pH25T minus CRM pH25T values were −0.0032 ± 0.0008 and −0.0060 ± 0.0019 pH units. Please note that this information in the cruise report^20^ is mistaken. No corrections were applied. To verify the precision of the pH measurements, replicate samples from the same Niskin bottle were analyzed twice during the cruise. The standard deviation of those analyses was ± 0.0015 pH units which could be considered as the reproducibility of the pH measurements during the TAlPro2016 cruise. During CRELEV2016 and ESAW, the precision of the measurements, evaluated from five determinations on subsamples collected from different Niskin bottles closed at the same depth, were 0.0004 and 0.0008 pH units, respectively.

### Total alkalinity data

During TAlPro2016, CO_2_ CRM (batch #147, the same as for DIC and pH analysis) samples were analysed to control the TA accuracy for every batch. As explained in^44^ and in the TAlPro2016 cruise report^20^, the final pH of every batch of analyses was corrected to match the certified CRM TA value on the corresponding analysis. In addition, any temporal drift within each batch of analyses was controlled by titrating filtered and stabilized surface seawater at the beginning and at the end of each batch. TA accuracy is ±2 μmol kg^−1^. Regarding the precision, due to time limitations, just once during the cruise replicate samples from the same Niskin bottle were titrated, the standard deviation of the 9 replicates was ±1 μmol kg^−1^. For CRELEV2016, some technical issues occurred at the electrodes of the VINDTA instrument, and many TA data were discarded. For the data that were kept, it has been estimated that the accuracy was ± 4 µmol kg^−1^, and the precision was <2 µmol kg^−1^. During ESAW, as estimated by the analysis of certified reference seawater (CRM, provided by A.G. Dickson, Scripps Institution of Oceanography, USA), the accuracy was better than ±4 μmol kg^−1^, and the precision was ± 2 μmol kg^−1^.

### Ion carbonate (CO_3_^2−^) data

The accuracy was not checked as the presence of mercury in the CRM bottles interferes with the lead reagent. Regarding the reproducibility, we analysed replicate samples collected from the same Niskin bottle, coming up with a standard deviation of ±6 μmol kg^−1^ (about ± 3% precision).

### Comments on quality control and internal consistency of CO_2_ data

During TAlPro2016 the laboratory temperature was high, varying from ≈30 °C in the first week to ≈27 °C in the second one, mainly due to calm weather, high air and water temperatures, and an air conditioning failure on board. Therefore, careful attention was paid to the temperature control in the thermostatic baths for DIC, pH and CO_3_^2−^. However, we recognise that the final quality of the whole CO_2_ system measurements is not ideal, as those in^44^. A first inspection of the CO_2_ measurements was done using typical oceanographical criteria, using property-to-property plots, with pressure and salinity being the independent variables for reference. Quality flags were assigned, using just three values, flag 2 for acceptable, 3 for non-acceptable and 9 for not measured or unavailable. An alternative way to check the precision, but not the accuracy, of the CO_2_ measurements is inspecting the coherence of the results in waters with a small temporal variability and/or high ventilation time scales, where a higher homogeneity is expected. The former conditions are difficult to find in the Mediterranean Sea where changes in deep and bottom waters were evidenced (and indeed are to be monitored by the Med-SHIP program). During TAlPro2016 we selected samples deeper than 2500 dbar in the Tyrrhenian Sea, where physical and chemical vertical gradients are small and the transient tracer concentrations are low, finding standard deviations of concentrations that compared well with the typical precision analysis from replicate samples (if available) given in the above sections.

The lack of CRM for pH measurements and difficulties in pH metrology to relate measured pH to a traceable International System of Units leads to a confusion between pH accuracy and precision. Consequently, internal consistency studies are usually used for a general estimation of the CO_2_ precision and accuracy. Recent publications deal with the lack of consistency between DIC, TA and pH, especially in regions, or waters with low pH^99^. There is no clear consensus on the reasons behind this mismatch^99,100^: pH method details regarding the equipment, the dye characterization (purified or unpurified), equation for pH calculation and corresponding valid ranges of temperature and salinity. In addition, several CO_2_ dissociation constants are available with no clear definition of the best recommended. A recent publication^101^ recommends^97^ refitted by^98^, or^102^ combined with the total boron concentration from^103^, in combination with recommendations in^44^ for the Mediterranean Sea. Using those constants, the pH, DIC and TA mean and standard deviation residuals (measured – calculated) values are respectively, −0.0029 ± 0.0085 pH units, −1.7 ± 5.1 μmol kg^−1^, 1.9 ± 5.5 μmol kg^−1^ for the whole water column and −0.0031 ± 0.0062 pH units, −1.8 ± 3.7 μmol kg^−1^, 1.9 ± 3.9 μmol kg^−1^ for deep Tyrrhenian Sea waters. Therefore, given the thresholds for consistency in GLODAP^18^ (i.e., 0.01–0.02 pH units for pH, and 4 μmol kg^−1^ for DIC and TA), the TAlPro2016 CO_2_ system measurements can be considered internally consistent.

Regarding CO_3_^2−^, the original spectrophotometric method by^72^ using PbCl_2_ evolved to^104^ with a small readjustment in the equations relating absorbance measurements and the final concentration referred to at 25 °C. Later^73^, changed the reagent to Pb(ClO_4_)_2_ with new equations. More recently^105^, readjusted the equations correcting inaccuracies in the wavelength accuracy of the spectrophotometer equipment. Finally^106^, widens the range of salinity and temperature equations validity. Those methods claim to be precise and accurate to about 2% the expected concentration. The scarcity of groups using these methods makes any assessment of a best method choice rather unclear. The CO_3_^2−^ concentrations were calculated and reported with^73^ equations, but the absorbance ratios and reference temperature can be used with more recent equations if required. With the same mentioned CO_2_ constants, the mean and standard deviation of measured minus calculated CO_3_^2−^ is 35 ± 14 μmol kg^−1^, with a clear increase of the residuals with increasing calculated CO_3_^2−^. Deep Tyrrhenian Sea water residuals are 38 ± 6 μmol kg^−1^, so the measurements seem to be neither accurate nor precise nor internally consistent with the other CO_2_ measurements. Further elaborating on this result is out of the scope of the current manuscript and is commented in a review work about the CO_3_^2−^ spectrophotometric technique^107^.

### Dichlorodifluoromethane and sulfur hexafluoride data

Standardization was performed by injecting small volumes of gaseous standard containing SF_6_ and CFC-12. This working standard was prepared by the company Dueste-Steiniger (Germany). The concentrations in the standard have been calibrated vs. a reference standard obtained from R.F. Weiss group at Scripps Institution of Oceanography, and the CFC-12 data are reported on the SIO98 scale. One calibration curve was measured to characterize the non-linearity of the system, and point calibrations were always performed between stations to determine the short-term drift of the detector. For TalPro2016, 19 replicate measurements were taken on 11 stations and the determined values for precision are 2.8% for SF_6_ and 1.0% for CFC-12. During CRELEV2016 and ESAW2016 no replicate measurements were done, but it is likely that precision is similar, given that samples were measured by the same operators and instruments. During ESAW2015 the Niskin bottles had rubber springs that caused a large contamination for SF_6_, so that all those samples had to be discarded.

### Thermosalinograph underway data

The SBE21 thermosalinograph that was used during CRELEV2016 was calibrated in January 2016. The SBE38 and SBE21 that were used during TAlPro2016 were calibrated in July 2008 and in January 2016, respectively. Initial accuracies for these two models are conductivity ± 0.001 S/m, temperature ± 0.01 °C (SBE21) and ± 0.001 °C (SBE38). The Turner fluorometer that was connected to the system was also calibrated in January 2016. To further validate temperature data, we made a graphic check of the horizontal surface patterns with sea surface temperature data (see Fig. 3), as well as checking for any noise or spikes in the underway data.

### Vessel-mounted acoustic doppler current profiler data

We checked and calibrated the underway data from ADCP using bottom-tracking methods to correct for ship motion and heading errors. Typical velocity accuracy is ±1.0% of measured velocity, or ±0.5 cm/s, for the Ocean Surveyor, and ±0.5% of measured velocity, or ±0.5 cm/s, for the WorkHorse Mariner. For CRELEV2016 the ADCP data have been post-processed with the CASCADE software package^108^, while for TAlPro2016 and ESAW the post-processing has been done with the CODAS Software System (https://currents.soest.hawaii.edu/docs/adcp_doc/), which allows extracting data, assigning coordinates, editing and correcting velocity data. Moreover, the data were corrected for errors in the value of sound velocity in water, and misalignment of the instrument with respect to the axis of the ship (about 0.3° for TAlPro2016, 1° for ESAW). After additional editing, to eliminate suspect values, and those where certain thresholds were surpassed, the output files were created by CODAS.

## Usage Notes

The aim of the paper is to provide an exhaustive description of the datasets that were collected during Med-SHIP 2016 cruises. It is thus not the aim to go into detail on oceanographic and biogeochemical aspects that came out from the data analysis. These have been (and will be further in future) the aims of specific scientific papers (e.g.^21,22,79,109,110^). The reader is invited to refer to those sources to get insight into the quality of a key multidisciplinary database that sets the baseline for future repetitions in the framework of the Med-SHIP initiative.

## Data Availability

No code was used to generate this dataset. Figure 5 was plotted by using Ocean Data View software (odv.awi.de), while the others have been generated using MATLAB ©.
